# Cardiac iron overload in chronically transfused patients with thalassemia, sickle cell anemia, or myelodysplastic syndrome

**DOI:** 10.1371/journal.pone.0172147

**Published:** 2017-03-03

**Authors:** Mariane de Montalembert, Jean-Antoine Ribeil, Valentine Brousse, Agnes Guerci-Bresler, Aspasia Stamatoullas, Jean-Pierre Vannier, Cécile Dumesnil, Agnès Lahary, Mohamed Touati, Krimo Bouabdallah, Marina Cavazzana, Emmanuelle Chauzit, Amandine Baptiste, Thibaud Lefebvre, Hervé Puy, Caroline Elie, Zoubida Karim, Olivier Ernst, Christian Rose

**Affiliations:** 1 Pediatrics Department, Necker Children’s Hospital, Assistance Publique-Hôpitaux de Paris, Paris, France; 2 Laboratory of Excellence GR-Ex, Paris, France; 3 Biotherapy Department, Necker Children’s Hospital, Assistance Publique-Hôpitaux de Paris, Paris, France; 4 Biotherapy Clinical Investigation Center, Groupe Hospitalier Universitaire Ouest, Assistance Publique-Hôpitaux de Paris, INSERM, Paris, France; 5 Hematology Department, Hôpital d’Adultes du Brabois, Vandoeuvre les Nancy, France; 6 Centre Henri Becquerel, Rouen, France; 7 Pediatric Oncology and Hematology Unit, Hôpital Charles Nicolle, Rouen, France; 8 Department of Biochemistry, Hôpital Charles Nicolle, Rouen, France; 9 Service d’Hématologie Clinique et de Thérapie Cellulaire, CHU, Limoges, France; 10 Service des Maladies du Sang, Hôpital Haut-Levêque, Pessac, France; 11 Paris Descartes-Sorbonne Paris Cité University, Imagine Institute, Paris, France; 12 INSERM UMR 1163, Laboratory of Human Lymphopoiesis, Paris France; 13 Département de Pharmacologie clinique et toxicologique, CHU, Bordeaux, France; 14 Paris Descartes Clinical Research Unit, Necker Children’s Hospital, Assistance Publique-Hôpitaux de Paris, Paris, France; 15 INSERM UMR 1149/ERL. CNRS 8252, Centre de Recherche sur l’inflammation, Paris, France; 16 French center for Porphyria, Louis Mourier Hospital, Assistance Publique-Hôpitaux de Paris, Colombes, France; 17 Radiology Department, Hopital Huriez, CHRU, Lille, France; 18 Hématologie clinique, Hôpital Saint Vincent de Paul, Université Catholique de Lille, Lille, France; Université Claude Bernard Lyon 1, FRANCE

## Abstract

The risk and clinical significance of cardiac iron overload due to chronic transfusion varies with the underlying disease. Cardiac iron overload shortens the life expectancy of patients with thalassemia, whereas its effect is unclear in those with myelodysplastic syndromes (MDS). In patients with sickle cell anemia (SCA), iron does not seem to deposit quickly in the heart. Our primary objective was to assess through a multicentric study the prevalence of cardiac iron overload, defined as a cardiovascular magnetic resonance T2*<20 ms, in patients with thalassemia, SCA, or MDS. Patient inclusion criteria were an accurate record of erythrocyte concentrates (ECs) received, a transfusion history >8 ECs in the past year, and age older than 6 years. We included from 9 centers 20 patients with thalassemia, 41 with SCA, and 25 with MDS in 2012-2014. Erythrocytapharesis did not consistently prevent iron overload in patients with SCA. Cardiac iron overload was found in 3 (15%) patients with thalassemia, none with SCA, and 4 (16%) with MDS. The liver iron content (LIC) ranged from 10.4 to 15.2 mg/g dry weight, with no significant differences across groups (*P* = 0.29). Abnormal T2* was not significantly associated with any of the measures of transfusion or chelation. Ferritin levels showed a strong association with LIC. Non-transferrin-bound iron was high in the thalassemia and MDS groups but low in the SCA group (*P*<0.001). Hepcidin was low in thalassemia, normal in SCA, and markedly elevated in MDS (*P*<0.001). Two mechanisms may explain that iron deposition largely spares the heart in SCA: the high level of erythropoiesis recycles the iron and the chronic inflammation retains iron within the macrophages. Thalassemia, in contrast, is characterized by inefficient erythropoiesis, unable to handle free iron. Iron accumulation varies widely in MDS syndromes due to the competing influences of abnormal erythropoiesis, excess iron supply, and inflammation.

## Introduction

Cardiac iron overload is the leading cause of death in patients with thalassemia who require chronic transfusion [1]. Cardiac iron overload has also been reported in a minority of patients with myelodysplastic syndromes (MDS) [2–4]. Iron overload has been associated with an increased risk of progression to leukemia and with shorter survival in non-chelated low-risk patients with MDS [4, 5]. The heart does not seem to be an early target for iron accumulation in chronically transfused patients with sickle cell anemia (SCA) [6]. Nevertheless, cardiac iron overload has been reported in a small percentage of chronically transfused young adults with SCA [7]. Hypotheses put forward to explain that the heart is relatively spared in SCA include a later onset of chronic transfusion compared to thalassemia, the use of erythrocytapheresis rather than simple transfusion, chronic inflammation that sequesters iron within reticuloendothelial cells, and efficient erythropoiesis capable of handling the iron from both transfused blood and hemolysis [6–10].

Blood transfusion is increasingly used in patients with MDS or SCA [11], who nevertheless are not routinely screened for iron overload and receive less iron chelation compared to patients with thalassemia [12]. These patients may, therefore, be at risk for iron-related comorbidities.

The primary objective of this study was to assess the prevalence of cardiac iron overload, defined as a cardiovascular magnetic resonance T2* <20 ms, in patients with thalassemia, SCA, or MDS. We also sought to identify factors associated with cardiac iron overload.

## Patients and methods

The study was approved by the appropriate ethics committee (Comité de Protection des Personnes d’Ile de France 2) (#21/21/2010) and supported by the AP-HP Department for Clinical Research and Development (DRCD), Paris, France. Written informed consent was approved by the ethics committee; it was obtained from each patient and/or both parents before study inclusion.

### Patients

Inclusion criteria were availability of a complete blood transfusion record, history of receiving at least 8 erythrocyte concentrates (ECs) in the past year, age older than 6 years to allow magnetic resonance imaging (MRI) without sedation, and affiliation to an health insurance system. Patients with heart diseases due to causes other than iron overload were not included. The patients were included between March 14, 2012, and February 21, 2014, at nine centers in France.

### Data collection

Iron accumulation in the heart and liver was assessed using MRI. Myocardial T2* (ms) and liver iron content (LIC) (mg of iron/g dry weight) were recorded. Within 6 months before or after MRI, blood was drawn for serum assays of iron, ferritin, non-transferrin-bound iron (NTBI), and hepcidin. NTBI was measured using the FeROSTM eLPI kit and hepcidin by liquid chromatography-tandem mass spectrometry. In patients treated with deferasirox, the drug was assayed in plasma using high performance liquid chromatography coupled with tandem mass spectrometry, as previously described [13].

The following data were collected retrospectively from the medical files of each patient: diagnosis, age at study inclusion, ethnic origin, history of splenectomy, serological status for hepatitis B and C (HCV), age at first transfusion, number of ECs transfused since the diagnosis, number of ECs transfused each year, time since initiation of chronic transfusion, transfusion procedure (simple transfusion, manual exchange transfusion, or erythrocytapheresis), age and ferritin levels at the beginning of chelation, and type of chelation.

Given the scarcity of published data on risk factors for cardiac iron overload in chronically transfused patients, we had no point of reference for estimating the sample size. We estimated that including 50 patients in each diagnostic group would allow us to identify associations linking selected parameters to cardiac iron overload.

### Imaging data

All patients underwent prospectively cardiac T2* by the same MRI method according to Anderson et al [14], liver iron content (LIC) according to the MRI method of Gandon et al [15], with 1.5 Tesla Machine and were validated in routine practice, evidencing the comparability of the measurements in the different centers [16].

### Statistical tests

Statistical analyses were performed using R software version 2.11.1. Descriptive statistics were computed for each diagnostic group. Continuous variables were described as mean ± SD if normally distributed and as median [min-max] otherwise. Categorical variables were described as n (%).

We used the chi-square test or Fisher’s exact test, as appropriate, to test for associations between categorical variables. To compare distributions of continuous outcomes across groups, ANOVA or the nonparametric Kruskal-Wallis test (when distribution was not normal) was performed. When the results indicated a significant overall difference, the *t* test or Wilcoxon test was applied to each pair of groups, and corresponding *P* values were adjusted using the Benjamini-Hochberg method to take the multiple comparisons into account. Spearman’s rho and *P* value of its corresponding test were calculated to investigate correlations between continuous parameters.

All *tests* were two-sided. *P* values ≤0.05 were considered significant.

## Results

### Descriptive data

One hundred and four patients were included in this study. Eighteen of them were excluded from our analysis (n = 2 did not meet inclusion criteria (total number of ECs received unavailable), n = 16 did not undergo cardiac T2* MRI or its result could not be collected). Thus, 86 patients were analyzed: 20 with thalassemia, 41 with SCA, and 25 with MDS (Table 1). As expected, the patients with MDS were older than were the patients with thalassemia or SCA, and most of them (96%) were of European descent. In the thalassemia group, 70% of patients had a history of splenectomy, a significantly higher proportion than in the other groups (*P*<0.001). The 12% prevalence of positive HCV serology in the SCA group may be ascribable to the history of episodic blood transfusions in some SCA patients during their infancy in Africa.

**Table 1:** Main patient characteristics.

N (%) or mean±SD	Thalassemia	SCA^a^	MDS^b^	*P* value
**Number of patients**	20	41	25	
**Male gender**	10 (50%)	21 (51.2%)	17 (68%)	0.34
**Age (y) at MRI**^**c**^	27.5±13.9	22.9±13.5	69.5±10.4	<0.001
**Origin:**				
• Africa • Europe • North Africa • Asia • Caribbean • Other	• 2 (10%) • 8 (40%) • 7 (35%) • 2 (10%) • 0 (0%) • 1 (5.0%)	• 31 (75.6%) • 1 (2.4%) • 2 (4.9%) • 0 (0%) • 2 (4.9%) • 5 (12.2%)	• 0 (0%) • 24 (96%) • 1 (4%) • 0 (0%) • 0 (0%) • 0 (0%)	
**History of splenectomy**	14 (70.0)	6 (14.6%)	2 (8%)	<0.001
**Positive HCV^**d**^ serology**	1 (5.9%)	5 (13.9%)	0 (0%)	0.45

^a^SCA, sickle cell anemia.

^b^MDS, myelodysplastic syndrome.

^c^MRI, magnetic resonance imaging.

^d^HCV, hepatitis C virus.

We divided the patients with SCA into two groups depending on whether their transfusion procedure at the time of the study was erythrocytapheresis (n = 11, E-SCA group) or another method (n = 30, including 29 given manual exchange transfusions and 1 simple transfusions, non-E-SCA group). The 25 patients with MDS were distributed as follows according to the WHO classification: refractory anemia with ring sideroblasts (RARS), n = 12; refractory anemia (RA), n = 6; unclassified, n = 2; refractory anemia with excess of blasts (RAEB-1), n = 2; 5q minus syndrome, n = 1; and myelodysplastic/myeloproliferative disease, n = 2. According to the International Prognostic Scoring System (IPSS) [17], they were all low risk: low risk in 15 cases and intermediate 1 in 10 cases.

Table 2 describes the main transfusion and chelation characteristics. Since MDS affects older patients, their age at transfusion initiation was significantly older, and their time on chronic transfusion and total number of ECs received since the diagnosis were significantly lower (*P*<0.01). When we compared the number of ECs given per year across groups, we found that the only significant difference was a higher number/year in the E-SCA group compared to the non-E-SCA group (35/y vs 21/y, *P* = 0.03).

**Table 2:** Transfusion and chelation data.

	Thalassemia	non-E-SCA^a^	E-SCA^b^	MDS^c^	*P* value
	N = 20	N = 30	N = 11	N = 25	
**Age at transfusion initiation (y)**	8.5 [0-45]	7 [0-45]	16.5 [1-55]	66 [38-83]	<0.001
**Time on chronic transfusion (y)**	10 [1-39]]	7 [1-22]	10.5[0-25]	3 [1-10]	<0.001
**Number of ECs**^**d**^ **since diagnosis**	359 [21-1360]	139 [24-791]	301 [14-888]	77 [16-544]	<0.001
**Number of ECs**^**d**^**/y**	24 [8-67]	21 [4-62]	35 [17-58]	27 [7-65]	0.09
					non-E-SCA
					vs. E-SCA
					= 0.03
**Number of patients given chelation**	19 (95%)	27 (90%)	8 (72.7%)	18 (72%)	0.10
**Age at chelation initiation (y)**	11 [1-48]	9 [2-47]	18 [6-31]	68 [38-84]	<0.001
**Ferritin at**	1148 [713-2400]	2075 [448	1500 [905-2804]	2398 [482	0.22
**chelation initiation**	(n = 8)	-3670]	(n = 5)	-5140]	
**(ng/mL)**		(n = 16)		(n = 12)	

^a^non-E-SCA, patients with sickle cell anemia given manual exchange transfusions (n = 19) or simple transfusions (n = 1).

^b^E-SCA, patients with sickle cell anemia managed with erythrocytapheresis.

^c^MDS, myelodysplastic syndrome.

^d^EC, erythrocyte concentrate.

Chelation was given to 95% of patients in the thalassemia group and 90% of those in the non-E-SCA group, compared to only 72% of patients in the E-SCA group and 72.7% of those in the MDS group. Age at chelation initiation was older in the E-SCA group than in the non-E-SCA group (18 vs. 9 years); the serum ferritin level at chelation initiation was apparently lower in the E-SCA group (1500 ng/mL vs. 2075 ng/mL in the non-E-SCA group), but this variable was missing for nearly half the patients and, therefore, statistical testing was not performed).

Data on chelation were available for 72 of the 86 patients. They are reported in Table 3. Seven patients received no chelation. Of the remaining 65 patients, 55 received deferasirox (14/20 with thalassemia, 25/41 with SCA, and 16/25 with MDS), 9 deferiprone (3/20 with thalassemia and 6/41 with SCA), and 1 deferoxamine. In each diagnostic group, about half the patients given deferasirox had at least one plasma deferasirox assay. Non-adherence to the deferasirox regimen, defined as a plasma level <0.5 μg/mL was found in none of the patients with MDS, 37.5% of patients with thalassemia, 30% of non-E-SCA patients, and 60% of E-SCA patients.

**Table 3:** Chelators used.

	Thalassemia	non-E-	E-SCA^b^	MDS^c^	Total
		SCA^a^ N = 30	N = 11	N = 25	
	N = 20				
**Intravenous Deferoxamine**	**1 (5%)**	**0 (0%)**	**0 (0%)**	**0 (0%)**	**1**
**Deferiprone**	**3 (15%)**	**5 (16.7%)**	**1 (9.1%)**	**0 (0%)**	**9**
**Deferasirox**	**14 (70%)**	**19 (63.3%)**	**6 (54%)**	**16 (64%)**	**55**
**No chelation**	**1 (5%)**	**3 (10%)**	**1 (9.1%)**	**2 (8%)**	**7**
**Unknown**	**1 (5%)**	**3 (10%)**	**3 (27%)**	**7 (28%)**	**14**

^a^non-E-SCA, patients with sickle cell anemia managed with manual exchange transfusions or simple transfusions.

^b^E-SCA, patients with sickle cell anemia managed with erythrocytapheresis

^c^MDS, myelodysplastic syndrome.

Table 4 reports the data on blood iron variables, LIC, and T2*.

**Table 4:** Blood iron variables, liver iron content, and T2*.

	ThalassemiaN = 20	non-E-SCA^a^	E-SCA^b^	MDS^c^N = 25	*P* value
		N = 30	N = 11		
N with T2*<20 ms	3 (15%)	0 (0%)	0 (0%)	4 (16%)	0.047
**LIC**^**d**^ **(mg/g dry weight)**	10.4 [0.8-20.2]	10.7 [0.8-37.1]	14 [0.8-19.7]	15.2[3.0-45.3]	0.29
**Plasma iron**	36.9 [31-57]	22.5 [6-45.2]	21 [13-46]	38.2 [11.9-72]	<0.001
**(μmoL/L)**					
**NTBI**^**e**^ **(μmoL/L)**	7.1 [0-31.1]	0 [0-18.3]	0 [0-12.4]	4.45 [0-25.5]	<0.001
**Ferritin (ng/mL)**	870	2739	2404	1611	0.08
	[169-4339]	[393-5596]	[33-20 030]	[223-6813]	
**Hepcidin (ng/mL)**	1.35 [0-12.3]	9.95 [0-67.9]	2.10 [0-52.4]	36.35 [3-143.2]	<0.001
**Ratio Hepcidin/LIC**	0.10 [0-2.40]	1.19 [0-8.48]	0.17 [0-3.11]	2.77 [0.18-	<0.001
**Median [range]**				19.61]	
**Ratio**	0.15 [0-0.93]	0.58 [0-4.41]	0.04 [0-0.32]	1.78 [0.23-	<0.001
**Hepcidin/Ferritinx10**^**2**^				10.22]	
**Median [range]**					
**Deferasirox plasma level <0.5 μg/mL**	3/8 (37.5%)	3/10 (30.0%)	3/5 (60.0%)	0/11 (0.0%)	0.03

^a^non-E-SCA, patients with sickle cell anemia managed with manual exchange transfusions or simple transfusions.

^b^E-SCA, patients with sickle cell anemia managed with erythrocytapheresis.

^c^MDS, myelodysplastic syndrome.

^d^LIC, liver iron content.

^e^NTBI, non-transferrin-bound iron.

LIC was significantly elevated in all four groups (median, 10.4-15.2 mg/g dry weight; normal, 0.4-2.2 mg/dry weight), with no significant difference across groups at the time of the study (*P* = 0.29).

**Fig 1:**
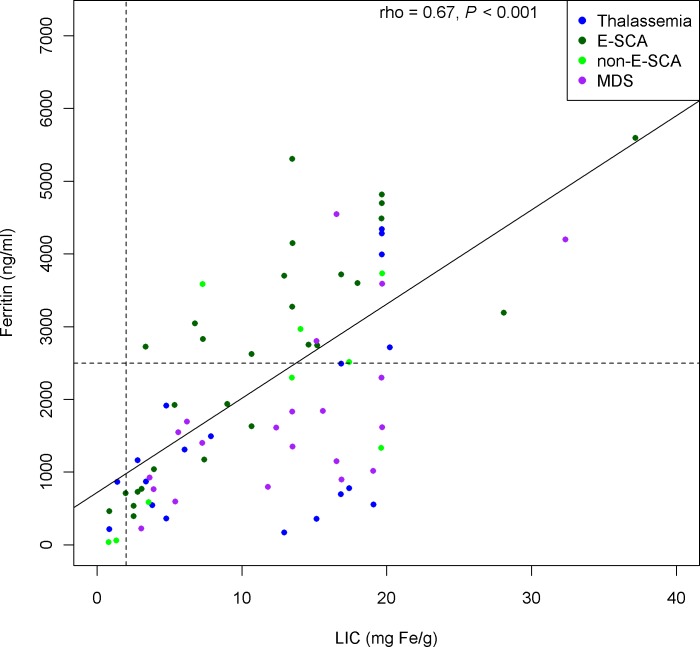
association between ferritin and liver iron content in the global population.

**Fig 2:**
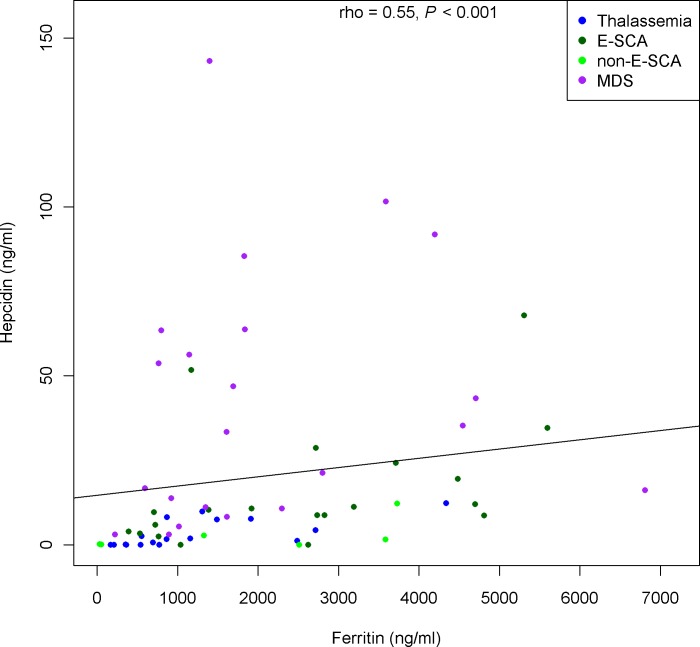
association between hepcidin and ferritin in the global population.

Cardiac iron overload defined as T2*<20 ms was diagnosed in 3 (15%) patients with thalassemia and 4 (16%) patients with MDS. No patient with SCA had T2*<20 ms, despite non-significantly higher serum ferritin levels (*P* = 0.08) in the two SCA groups. Ferritin levels were strongly associated with LIC both in the overall population and in each group (Fig 1). Serum iron was elevated in the thalassemia and MDS groups but normal in the SCA groups, producing a highly significant difference (*P*<0.001). NTBI was also high in the thalassemia and MDS groups and considerably lower in the SCA groups (*P*<0.001). The largest differences across groups were for the serum hepcidin levels, which were low in the patients with thalassemia, normal in those with SCA, and markedly elevated in those with MDS (*P*<0.001). NTBI was significantly associated with serum iron (rho = 0.46, p<0.001), but with none of the others parameters studied (ferritin, LIC, years of transfusion therapy, hepcidin level). On the whole population, hepcidin level was significantly associated with ferritin (rho = 0.55, p<0.01), LIC (rho = 0.35, p<0.01), and with years of transfusion therapy (rho = -0.31, p<0.01). The association between hepcidin level and ferritin was the only one that remained strong and significant in most of the disease groups (it was non significant for MDS) (Fig 2).

### Factors associated with cardiac iron overload

None of the patients with SCA had cardiac iron overload. LIC elevation was associated with cardiac iron overload in the patients with thalassemia (*P* = 0.04) or MDS (*P* = 0.03). Surprisingly, cardiac iron overload was not significantly associated with any of the transfusion or chelation parameters, but our study is limited by the short number of patients developing cardiac iron.

## Discussion

Over 300 000 children are born each year with major thalassemia or SCA. Cardiac iron overload is the leading cause of death in thalassemia. In the UK, routine MRI T2* monitoring and improved iron chelation have been associated with a 71% decrease in deaths due to thalassemia [18]. The relationship between excess iron and mortality in SCA is more difficult to analyze, because the patients with transfusion-related iron overload are also those who have greater disease severity requiring chronic transfusion [19, 20]. However, although additional long-term prospective studies are still needed [21], blood transfusion is increasingly used in patients with SCA, particularly for primary and secondary stroke prevention [22]. As a result, iron-related toxicity has been reported in SCA [7, 23]. Clearly, there is a pressing need for evaluating the potential consequences of transfusion iron overload in SCA. Finally, there is increasing evidence that iron chelation benefits patients with MDS [2–5], not only by diminishing the deposition of iron in the liver and heart, but also by alleviating the adverse effects of iron on erythroid precursors [3, 24].

In thalassemia, both the liver and the heart contain excess iron, whereas in SCA the iron deposits selectively in the liver. In a study of 141 patients with SCA who died in adulthood, at a mean age of 36±11 years, Darbari et al found that 16 (11.4%) had cirrhosis and 10 (7.1%) iron overload; seven of 16 (43.8%) patients with cirrhosis had iron overload compared with 3 of the 125 without cirrhosis (2.4%) (*P*<0.001) [23]. The heart seems less affected by iron overload in children and young patients [25–27], although iron-related cardiomyopathy has been reported in older patients. Thus, in a series of 201 chronically transfused patients with SCA, 6 had cardiac iron overload, including 1 aged 17 years and 5 aged 22 to 29.6 years [7]. We found no cardiac iron overload in our 41 patients with SCA, whose median age was 22.9±13.5 years, whereas LIC values in the SCA groups were similar to those in the thalassemia and MDS groups. These results confirm that, in patients with SCA, iron overload selectively targets the liver, leaving initially the heart relatively spared.

Our finding of severe iron overload with LIC elevation in patients with SCA managed using erythrocytapheresis contrasts with previous reports [10, 28, 29]. Several factors may explain this discrepancy. First, duration of transfusion was longer in our patients than in previous studies. Given the severity of the anemia in the SCA patients included in this study, the centers programmed each automated erythrocytapheresis procedure to increase the hemoglobin level by 1.5 g/dL compared to the pre-procedure value, which can induce substantial accumulation of iron over several years. Second, adherence to chelator therapy seemed low in the E-SCA group: of the 5 patients with plasma deferasirox assays, 3 had low values. Physicians may direct insufficient attention to adherence because of a misconception that erythrocytapheresis prevents totally iron overload. Efforts are needed to raise physician awareness about the risk of iron overload despite erythrocytapheresis.

The 16% prevalence of cardiac iron overload in the MDS group is consistent with earlier data from chronically transfused patients with MDS (18% [30] and 16% [31]). Cardiac iron overload may contribute to heart failure in this older population, in addition to chronic anemia and comorbidities.

Liver iron overload has been reported to antedate cardiac iron overload in patients with thalassemia or MDS [32, 33]. Cardiac iron overload usually develops only after severe hepatic iron overload but not exclusively, since some patients may accumulate iron in heart whereas they have a negative liver iron balance [33]. In spite of the low frequency of cardiac iron overload in our cohort, resulting in a lack of power to detect associations, we found a significant relationship between increased LIC and abnormal T2* in patients with thalassemia (*P* = 0.04) and MDS (*P* = 0.03). Some other studies found poor correlation, this discrepancy might be be explained by differences in the kinetics of iron uptake and in iron elimination in the heart and the liver [33].

Cardiac iron overload as defined by T2*<20 ms was absent in SCA but present in some patients with thalassemia or MDS despite similar total numbers of ECs received. The reasons for this finding are unclear. The efficiency of erythropoiesis may play a key role in determining the rate and location of iron deposition. In diseases characterized by inefficient erythropoiesis, whether inherited such as thalassemia or acquired such as MDS, hepcidin production is suppressed and iron absorption increased [34, 35]. Thus, our patients with thalassemia had low hepcidin levels, high serum iron and NTBI, and more organ damages, since iron under NTBI form enters the myocytes through the voltage-dependent L-type calcium channels and induces the development of a cardiomyopathy [36]. Hepcidin levels were normal in SCA patients, whose low NTBI levels resulted probably from massive consummation of iron through effective erythropoiesis, making NTBI less available in the circulation. A recent study compared 5 patients with thalassaemia, 5 with SCA, and 5 with Diamond-Blackfan anemia [37]. The three groups were comparable for age, time on chronic transfusion, and time on chelation. NTBI levels were lowest in the SCA group, suggesting better iron utilization. Further support for this explanation comes from a study of iron overload in 121 chronically transfused children with SCA enrolled in the TWITCH trial [38]. Interestingly, despite a mean time on chronic transfusion of 4.1±2.4 years, mean transferrin saturation was 47.2%±23.6%, i.e., lower than the value expected after a comparable transfusion time in patients with thalassemia. We did not collect transferrin saturation data. However, serum iron reliably mirrors transferrin saturation in patients without hypotransferrinemia. In our study, serum iron was normal in the patients with SCA. Conceivably, in SCA, the large amounts of heme-bound iron from transfused blood and hemolysis may be efficiently handled by the liver macrophages, transferred to the bone marrow, and used to produce erythrocytes, thereby preventing the formation of NTBI. Furthermore, chronic inflammation, which increases hepcidin levels, is a prominent feature of SCA that limits the release of iron release from macrophages [39]. A recent study of adults with SCA showed that the percentage of reticulocytes and levels of erythropoietin, ferritin, and C-reactive protein contributed to variations in hepcidin levels [40]. Hepcidin levels were very high in our patients with MDS. In previously published works, hepcidin levels varied across MDS subtypes [3], due to the conflicting influences of dyserythropoiesis, transfusion-related iron overload, and inflammation [41]. Our patients with MDS were heavily transfused and had high hepcidin levels, without major inflammation (median C-reactive protein, 5.0 mg/L [range, 0-42]), suggesting that iron overload in this population increased the production of hepcidin, albeit not sufficiently to prevent high transfusion-related NTBI release.

In conclusion, in SCA, the liver is the main target for iron accumulation, and the heart is usually initially spared, perhaps because iron is recycled by efficient erythropoiesis and trapped within macrophages due to chronic inflammation. Iron overload can occur in SCA even when erythrocytapheresis is used. In thalassemia, erythropoiesis is inefficient, allowing free iron to deposit within tissues. In MDS, the heterogeneity of the causative diseases and competing influences of abnormal erythropoiesis, iron overload, and inflammation produce widely variable results.
